# Increased cervical cancer incidence in the target age of screening—Variation by mode of detection

**DOI:** 10.1002/ijc.70371

**Published:** 2026-02-07

**Authors:** Jenna Snellman, Maiju Pankakoski, Sirpa Heinävaara, Veli‐Matti Partanen, Maija Vahteristo

**Affiliations:** ^1^ Faculty of Medicine University of Helsinki Helsinki Finland; ^2^ Department of Screening Registry Finnish Cancer Registry Helsinki Finland

**Keywords:** cervical cancer, cervical cancer risk factors, HPV testing, interval cancer, mode of detection

## Abstract

Cervical cancer incidence has increased in several high‐income countries over the last decades. During the same period, many screening programs have shifted their primary method from cytology to the more sensitive HPV test. To better understand the increasing incidence, we visualized the incidence trends by mode of detection (screen‐detected, interval cancers, cancers in non‐participants, or cancers in those outside screening target ages) and by cancer morphology type using segmented regression. We also assessed the effects of the primary test method on the risk of screen‐detected and interval cancers using Poisson regression. The study population consisted of all individuals diagnosed with cervical cancer (*n* = 4472) during 1996–2022 in Finland. An increase in cervical cancer incidence was observed during the 2010s, predominantly among individuals within the screening age. A clear upward trend was evident among both screening participants and non‐participants. Notably, the incidence of screen‐detected cervical cancer rose sharply after approximately 2013. The introduction of HPV‐based screening was associated with an increased detection rate of screen‐detected cancers (RR = 1.4, 95% CI = 1.1–1.7) compared to cytology testing. Conversely, the risk of interval cervical cancer was reduced when the preceding screening method was an HPV test (RR = 0.29, 95% CI = 0.21–0.38) compared to cytology. Our results imply that the initiation of HPV testing has improved the detection of prevalent cancers, but changes in risk factors also contribute to the rising incidence of cervical cancer.

## Graphical abstract

What's new?

Cervical cancer rates have risen in several high‐income countries despite advances in screening. Here, using data on cervical cancer diagnoses between 1996 and 2022 in Finland, the authors examined incidence trends according to detection mode—screening, between screenings, or outside screening programs—with attention to the role of human papillomavirus (HPV) testing. Analyses show that cervical cancer diagnoses increased in Finland during the 2010s, regardless of screening. After 2013, however, screen‐detected cervical cancer increased significantly, with HPV‐based testing fueling detection during routine screening. The findings point to HPV testing as a key tool for more effective cervical cancer screening.

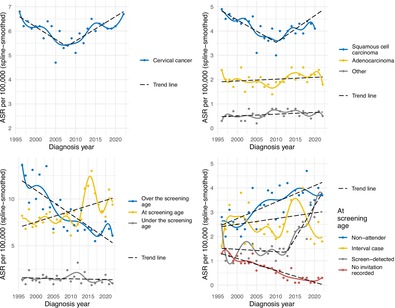

**Abbreviations**
APC: annual percentage changes
FCR: The Finnish Cancer Registry
HPV: human papillomavirus

## 1 INTRODUCTION

Screening has significantly reduced the incidence of cervical cancer in high‐income countries.^1^, ^2^, ^3^ However, recent data indicate an increasing incidence of cervical cancer among women aged 30–39 years in several high‐income countries, including the United Kingdom, Estonia, and Sweden.^4^, ^5^, ^6^ This rather abrupt change in incidence may be attributed to changes in organized screening programs, as well as increased exposure to cervical cancer risk factors.^2^, ^4^ Moreover, declining participation in screening programs may also contribute to the observed incidence increase.^7^, ^8^ Non‐participants are more likely to be diagnosed with cervical cancer than participants.^9^, ^10^

Mode of detection refers to the classification of cancer cases based on an individual's invitation status and participation in the screening program prior to cancer diagnosis. Based on this classification, cases can be categorized as screen‐detected cancers, interval cancers, cancers among non‐attendees, and cancers among the uninvited—that is, individuals outside the target population due to age or other eligibility criteria.^11^, ^12^, ^13^

Interval cancers, cancers diagnosed following a negative screening test or colposcopy but before the next screening round, indicate a potential failure in the screening program.^14^ To our knowledge, trends in cervical cancer incidence have not previously been assessed according to mode of detection. Such stratification may help reveal underlying factors contributing to the observed increase in incidence.

Almost all cases of cervical cancer are associated with high‐risk human papillomavirus (HPV) infection.^15^, ^16^, ^17^ HPV testing has become more common as a screening method since it provides better protection against cervical cancer compared to cytology.^18^ Since HPV testing is more sensitive compared to traditional cytology (i.e., Pap test), it could result in a reduction in interval cancers and an increase in screen‐detected cancers in the first screening rounds.^18^, ^19^ Furthermore, in international studies, HPV testing has shown better effectiveness than cytology testing, especially against adenocarcinomas.^18^ On the other hand, in the Finnish setting, the effectiveness of the test methods was similar in a long‐term follow‐up trial.^20^

The aim of this study was to deepen the understanding of the changes in the incidence of cervical cancer over the last decades. We examined cervical cancer incidence by the mode of detection with regards to screening and by morphology type during 1996–2022 in Finland. We also examined the effect of the primary test method on screen‐detected and interval cancers.

## 2 MATERIALS AND METHODS

Our register‐based study population consisted of all individuals diagnosed with the first primary cervical cancer (ICD‐O‐3: C53)^21^ during the study period from 1996 to 2022. Data on diagnosed cancer cases were received from the Finnish Cancer Registry (FCR), which has comprehensive cancer data on solid tumors, with completeness estimated at 96%.^22^ The individual cancer registry data were then linked with the data on the individual's screening history from the screening registry also maintained by FCR. These data consisted of complete information on invitations to organized screening and screening tests.

During the study period, municipalities in Finland were responsible for organizing cancer screening for their residents. Although the official target age for screening was 30–60 years, some municipalities also invited 25‐ and/or 65‐year‐olds. The invitation coverage of 25‐year‐olds ranged between about 25% and 35% until 2018, but after that, it increased to over 40%. For 65‐year‐olds, the coverage was approximately 15% until 2018, increasing to about 40% thereafter.^5^ Since 2022, 65‐year‐olds throughout the country have been invited. The target group receives a screening invitation every 5 years. In case of a mild abnormal result (HPV‐positive with triage cytology result of negative or atypical squamous cells of undetermined significance in HPV‐based testing, and atypical squamous cells of undetermined significance in cytology‐based testing), the person receives a new screening invitation after 1–2 years. More severe results warrant a referral to colposcopy.

The Finnish national screening program has gradually shifted from primary cytology to primary HPV testing from 2003 onwards.^23^ The wider initiation of primary HPV testing began around 2012. During the study period, the proportion of HPV tests in the program gradually expanded but was less than 20% until 2018. In 2019, HPV testing was introduced widely and used in over 60% of all primary tests, and in 2022, the share of HPV tests was 75% (Supplement Figure S1).^5^, ^24^

The six modes of cancer detection in our study were formed based on the European screening guidelines.^13^

1. Screen‐detected cancers: cases with a prior screening test and referred to colposcopy and biopsy up to 12 months before diagnosis.
2. Interval cancers: cases with a prior screening test recorded up to 5.5 years before diagnosis but no referral to colposcopy and biopsy or cases with referral to colposcopy and biopsy but diagnosis more than 12 months after the screening visit.
3. Non‐attenders: cases of whom a prior screening invitation was recorded up to 5.5 years before diagnosis without attendance.
4. No screening invitation was recorded: cases for which no invitation was recorded, although they were of screening target age. This was mainly related to problems with registration.
5. Under the screening target age: cases did not receive an invitation because they were younger than the screening target age
6. Over the screening target age: cases had no invitation because they were older than the screening target age

We examined the annual incidence of cervical cancer from 1996 to 2022 by calculating age‐standardized incidence rates per 100,000 by cancer morphology type and by mode of detection using direct age‐standardization. We used the Finnish standard population in 2014 and five‐year age groups in the standardization. Population denominators in the mode‐specific analyses were defined in the following manner:

1. Under the screening target age: women below age 25, plus the proportion of 25–29‐year‐olds not invited each year.
2. At screening age: women aged 30–64 years, plus the proportion of 25–29‐ and 65–69‐year‐olds invited each year.
3. Over the screening target age: women above age 64, plus the proportion of 65–69‐year‐olds not invited each year.

We used splines to smooth the curves in the incidence curve visualizations. Additionally, we modeled the incidence rates with segmented regression to analyze the trends by detection mode and morphology status. Separate segmented regression models were fitted on each incidence curve using the R package *segmented*.^25^ We calculated segmented annual percentage changes (APC) separately for each segment identified with the regression models using the model slopes, assuming constant growth.^26^ If no breakpoints were detected, APC was calculated for the full study period.

We examined the impact of the screening test method on the risk of interval and screen‐detected cancers in 2003–2022. This was done by defining whether the test preceding the diagnosis was conducted using a Pap test or an HPV test. Separately for both types of tests, the number of screen‐detected and interval cancers preceded by the testing were divided by the number of all tests conducted between 2003 and 2022. These percentages were reported along with 95% confidence intervals. This analysis was further broken down into five‐year periods to get an overview of the temporal trend. In addition, risk ratios with 95% confidence intervals for the effect of the test method on screen‐detected and interval cancers during the whole period were calculated using Poisson regression, adjusting for diagnosis year. All analyses were performed using R version 4.3.3.

## 3 RESULTS

**FIGURE 1:**
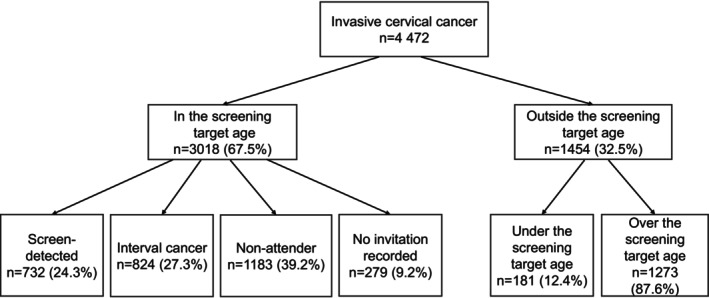
The first primary cervical cancer cases between 1996 and 2022 were divided into different groups by the mode of detection.

We had a total of 4472 cervical cancer cases reported between the years 1996 and 2022 in Finland. The median age at diagnosis was 51 years. The background characteristics of the cases are shown in Table 1. Of all cases, 62% were squamous cell carcinomas and 29% were adenocarcinomas. The cancer cases by detection mode are shown in Figure 1. Of the cancers, 68% were detected in individuals within the target age of screening. The single group with the highest number of cases was those over the screening target age (*n* = 1273).

**TABLE 1:** Background characteristics of 4472 cases in total.

Variables	*N*	%
Age		
10–19	5	0.1
20–29	251	5.6
30–39	995	22.2
40–49	919	20.6
50–59	661	14.8
60–69	579	12.9
70–79	586	13.1
80–89	396	8.9
90–99	80	1.8
Morphology		
Squamous cell carcinoma	2769	61.9
Adenocarcinoma	1317	29.4
Other type	386	8.6
Number of cases		
1996–1998	497	11.1
1999–2001	488	10.9
2002–2004	484	10.8
2005–2007	435	9.7
2008–2010	442	9.9
2011–2013	484	10.8
2014–2016	530	11.9
2017–2019	527	11.8
2020–2022	585	13.1

**FIGURE 2:**
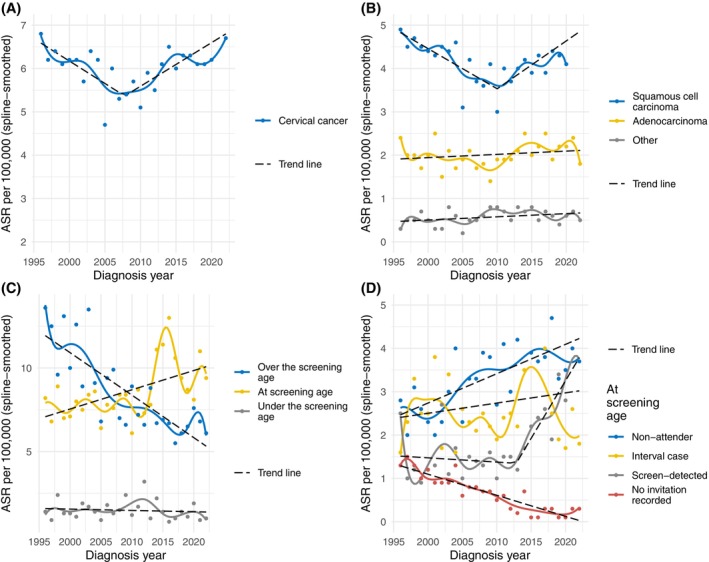
Age‐standardized cervical cancer incidence rates (ASR) per 100,000 in 1996–2022 with segmented regression lines: (A) all cervical cancers, (B) by morphology type, (C) by age group, and (D) by detection mode in the screening target age.

Figure 2 presents cervical cancer incidence rates in 1996–2022 overall, by morphology, and by different detection modes. There was a decline in the overall cervical cancer incidence in 1996–2007 (APC = −1.72; 95% CI = −2.87, −0.57) after which the trend was upward during 2008–2022 (APC = 1.65; 95% CI = 0.84, 2.46) (Figure 2A, see Table S1 for the estimates).

The decline in incidence in 1996–2009 was particularly associated with squamous cell carcinomas (APC = −2.19; 95% CI = −3.35, −1.03) (Figure 2B). The gradual increase in cervical cancer incidence since 2010 was mostly attributed to individuals of screening age (APC = 1.34; 95% CI = 0.52, 2.16), whereas incidence among individuals over the screening target age (Figure 2C) decreased quite steadily during the study period (APC = −2.95; 95% CI = −3.84, −2.06). Incidence among individuals under the screening target age remained quite stable over the years with small annual variation.

Figure 2D shows a closer breakdown of the cancer cases among individuals at screening age. A clear upward trend in the incidence was visible in the screening attendees and non‐attendees. The increase in the incidence of screen‐detected cases was particularly steep in 2013–2022 (APC = 10.27; 95% CI = 6.02, 14.53), whereas the incidence in the non‐attenders grew steadily throughout the study period (APC = 2.02; 95% CI = 1.09, 2.95). Interval cancer incidence showed more annual variation compared to the other groups. Overall, the interval cancer incidence increased slightly, but not significantly, during the study period (APC = 0.86; 95% CI = −1.32, 3.06). The figure also shows a steady decline in the incidence of cancers with no screening invitation recorded (APC = −7.37; 95% CI = −8.78, −5.96).

**FIGURE 3:**
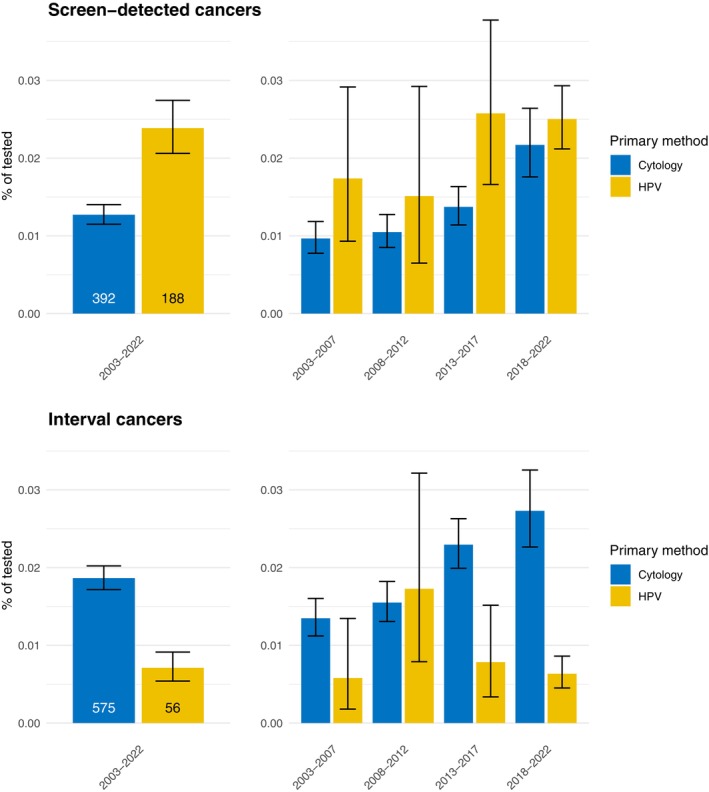
The percentages of screen‐detected and interval cancers in 2003–2022 by test method in the preceding screening visit. The number of cancer cases in each group is annotated in the graph, as well as 95% confidence intervals for the percentages.

Figure 3 shows a further breakdown of the interval and screen‐detected cases by test method in the preceding screening visit, for the years 2003–2002 and in 5‐year periods. The proportion of both interval and screen‐detected cancers differed significantly by primary test method. HPV testing was associated with an increased probability of screen‐detected cancer (RR = 1.4, 95% CI = 1.1–1.7) compared to cytology testing. Conversely, the probability of an interval cancer decreased if the preceding screen was performed with an HPV test (RR = 0.29, 95% CI = 0.21–0.38). Interval cancers preceded by HPV testing were especially uncommon in the latest years of the follow‐up.

## 4 DISCUSSION

In our study, the gradual increase in cervical cancer incidence observed from approximately 2010 onward was mostly attributable to individuals within the screening age range. Notably, the incidence of screen‐detected cancers rose sharply after around 2013. Compared to cytology‐based testing, HPV testing was associated with a higher probability of cancers being detected through screening. In contrast, the probability of interval cancer was lower following a screening performed with an HPV test compared to cytology. Among individuals within the screening age range, a clear upward trend in cervical cancer incidence was observed in both screening attenders and non‐attenders. These findings suggest that the overall increase in incidence was, at least in part, driven by a rise in underlying cervical cancer risk factors rather than by substantial changes in screening participation. This interpretation is supported by the observation that overall screening attendance remained relatively stable at approximately 70% throughout the study period.^6^

The most well‐established and major risk factor for cervical cancer is persistent infection with high‐risk HPV.^15^, ^16^, ^17^ Other recognized risk factors include smoking, other sexually transmitted infections, low socioeconomic status, immunosuppression, long‐term use of oral contraceptives, and high parity.^27^, ^28^, ^29^, ^30^, ^31^, ^32^, ^33^, ^34^ Estimating HPV prevalence before the widespread implementation of HPV testing is challenging; however, HPV is considered the most common sexually transmitted infection.^15^, ^35^, ^36^ Changes in sexual behavior over past decades are believed to have contributed to an increased prevalence of the virus.^2^, ^29^, ^37^ The rising prevalence of oncogenic HPV genotypes has likely played a role in the observed increase in cervical cancer incidence.^38^ Rising obesity rates may also influence cancer progression.^39^, ^40^ Additionally, the use of oral contraceptives in Finland increased substantially during the 1980s, with the number of users doubling within a decade.^41^ Use of oral contraceptives for more than 5 years has been associated with a significantly elevated risk of cervical cancer.^32^, ^42^ Furthermore, changes in the management of precursor lesions may also have influenced cancer development. Prior to 2006, treatment was recommended for mild precursor lesions; however, the updated Finnish treatment guidelines introduced in 2006 recommended surveillance rather than immediate treatment for younger women, given the high likelihood of spontaneous regression.^43^

The overall incidence of screen‐detected cervical cancers increased, particularly over the past decade in Finland. When stratified by test method, HPV testing was associated with a higher probability of screen‐detected cancer compared to cytology. During the study period, HPV testing was gradually implemented in the national screening program.^5^, ^24^ The gradual implementation of HPV testing in Finland has likely increased the number of screen‐detected cancers temporarily among the HPV tested due to its higher sensitivity compared to cytological testing.^18^, ^19^ In trials, HPV testing has been shown to detect a higher number of cancers and precursor lesions during the initial rounds of screening.^44^ Although HPV testing offers greater sensitivity than cytology, it is less specific. As a result, it increases demands on healthcare resources and may elevate the risk of overdiagnosis and overtreatment due to a higher number of referrals for colposcopy.^45^

Conversely, we observed a significantly higher probability of interval cancers among individuals screened with cytology compared to those screened with HPV testing. This likely reflects the improved sensitivity of HPV testing, which enables earlier detection of precancerous lesions and cancers, resulting in a higher number of screen‐detected cases and fewer interval cancers.

HPV testing may detect adenocarcinomas more effectively compared to primary cytology.^18^ However, such a difference in long‐term effectiveness could not be found in a recent Finnish study.^20^ Overall, screening prevents squamous cell carcinoma better than adenocarcinoma.^18^, ^46^ After the gradual implementation of primary HPV testing since 2012, especially the incidence of squamous cell carcinoma increased, which could partly be due to better sensitivity of HPV testing for squamous carcinoma. The incidence of adenocarcinoma, however, did not show a meaningful increase. The overall cervical cancer incidence had begun to rise already a few years prior to the wider implementation of HPV testing, which highlights the role of the increased prevalence of risk factors in the population.

Cervical cancer incidence decreased during the follow‐up among those over the screening age. One plausible reason is the increased invitation coverage in the oldest age groups over time. The invitation coverage of 60–64‐year‐olds has increased since the 1990s,^6^ and the attendance rate of those who received an invitation has been over 70% in the oldest screening age groups.^5^, ^46^ The screening age was extended to include 65‐year‐olds in 2022 in Finland, since screening has been found to be effective in the older population, and since cancer cases are increasing in this group as the population of Finland is aging.^11^, ^12^, ^46^, ^47^ Additionally, the variation of screening histories may influence cervical cancer incidence in the older age groups.^48^ The younger cohorts of those over the screening age have had more opportunities to participate in cervical cancer screening during their lifetime, as the nationwide program was only established in the 1970s. In contrast, older cohorts had limited or no access to organized screening at comparable ages, which may contribute to higher incidence in those groups. Our findings support the appropriateness of the current screening age limits.

In Finland, cervical cancer screening is population‐based, with all eligible individuals invited. Screening aged individuals without data on invitation were mainly cases where screening likely occurred but was not registered due to data delivery issues. During the follow‐up, the proportion of individuals without a recorded invitation declined, as invitation coverage rose from around 90% in the 1990s to nearly 100% since the early 2000s.^24^ This also explains the technical decline in incidence in this group.

The strengths of this study include the population‐based register data with many years of follow‐up and the linkage of cancer diagnoses and screening data by using a unique personal identifier. In addition, the coverage of cancer registration was excellent throughout the study period. A limitation of this study is the lack of data on opportunistic cervical testing, which is common in Finland, especially among individuals under the target screening age and within the screening age group.^34^, ^46^, ^49^ However, many non‐attenders do not participate in opportunistic testing either. Nevertheless, some cancers in individuals below the screening target age and in non‐attenders may have been detected through screening‐type testing of asymptomatic individuals. In previous years, opportunistic testing was done by Pap testing, and its coverage did not differ between Pap‐ and HPV‐tested groups in the program. Therefore, it is unlikely that opportunistic testing would bias the comparison between these methods.^20^ It should also be noted that the HPV‐vaccinated individuals had not reached the screening age during the study period. In the future, vaccinations will likely reduce the prevalence of most oncogenic HPV infections and thus the number of cancers.

Based on our results, HPV testing has likely increased the number of screen‐detected cancers. It is essential to assess the effectiveness of HPV testing as its use becomes more common globally. Also, the use of cytology testing needs to be evaluated, and the quality of it monitored, for example in the form of auditing cancer cases or re‐reading cytological samples.^50^ Collecting temporal trend data on the prevalence of different HPV genotypes would be valuable for predicting future cancer burden. Our study suggests that changes in risk factors also contribute to the increasing incidence of cervical cancer. Which risk factors and how is an important question for future research.

To our knowledge, this is the first study to examine the long‐term cervical cancer incidence by the mode of cancer detection with regards to screening. Our results show that the incidence of cervical cancer is increasing among both screening attendees and non‐attendees. The current age limits for screening appear to be appropriate. The increase in screen‐detected cancers can be attributed to the wider adoption of HPV testing and the growing influence of risk factors. Based on our study, the recommendation for primary HPV testing in organized screening is justified.

## AUTHOR CONTRIBUTIONS

**Jenna Snellman:** Conceptualization; formal analysis; investigation; visualization; writing – original draft; writing – review and editing. **Maiju Pankakoski:** Conceptualization; data curation; formal analysis; investigation; methodology; visualization; writing – review and editing. **Sirpa Heinävaara:** Conceptualization; methodology; project administration; resources; writing – review and editing. **Veli‐Matti Partanen:** Conceptualization; writing – review and editing. **Maija Vahteristo:** Conceptualization; data curation; investigation; methodology; formal analysis; supervision; visualization; project administration; writing – review and editing.

## CONFLICT OF INTEREST STATEMENT

The authors declare no conflicts of interest.

## Supporting information


**Table S1.** Annual percentage change (APC) estimates and 95% confidence intervals.
**Figure S1.** The proportion of HPV tests in the screening program in 1996–2022.

## Data Availability

R code is publicly available on Github (https://github.com/FinnishCancerRegistry/original_research_analyses/blob/d6636d35071723d8a717429c03b05b1448cdd6b9/Increased%20cervical%20cancer%20incidence%20in%20the%20target%20age%20of%20screening%20%E2%80%93%20variation%20by%20mode%20of%20detection.R). Interested researchers may contact the Finnish Social and Health Data Permit Authority, Findata, for permission to use the data for scientific research under the Act on the Secondary Use of Social and Health Data. Further information is available from the corresponding author upon request.

## References

[1] Pedersen K , Fogelberg S , Thamsborg LH , et al. An overview of cervical cancer epidemiology and prevention in Scandinavia. Acta Obstet Gynecol Scand. 2018;97(7):795‐807. doi:10.1111/aogs.13313 29388202

[2] Anttila A , Pukkala E , Söderman B , Kallio M , Nieminen P , Hakama M . Effect of organised screening on cervical cancer incidence and mortality in Finland, 1963–1995: recent increase in cervical cancer incidence. Int J Cancer. 1999;83(1):59‐65. doi:10.1002/(SICI)1097-0215(19990924)83:1<59::AID-IJC12>3.0.CO;2-N 10449609

[3] Adegoke O , Kulasingam S , Virnig B . Cervical cancer trends in the United States: a 35‐year population‐based analysis. J Womens Health. 2012;21(10):1031‐1037. doi:10.1089/jwh.2011.3385 PMC3521146 22816437

[4] He WQ , Li C . Recent global burden of cervical cancer incidence and mortality, predictors, and temporal trends. Gynecol Oncol. 2021;163(3):583‐592. doi:10.1016/j.ygyno.2021.10.075 34688503

[5] Partanen V‐M , Heinävaara S , Sarkeala T , Pankakoski M . Kohdunkaulasyövän seulontaohjelma – vuosikatsaus 2023. July 2023. Accessed June 26, 2024. https://syoparekisteri.fi/assets/files/2023/07/Kohdunkaulasyovan-seulontaohjelman-vuosikatsaus_2023.pdf

[6] Finnish Cancer Registry . Cancer Statistics. Syöpärekisteri. Accessed June 26, 2024. https://cancerregistry.fi/statistics/cancer-statistics/

[7] Cervical Screening Programme, England – 2020‐21 [NS] . NHS England Digital. Accessed December 13, 2024. https://digital.nhs.uk/data‐and‐information/publications/statistical/cervical‐screening‐annual/england‐‐2020‐2021

[8] Aitken CA , Kaljouw S , Siebers AG , et al. Investigating the decrease in participation in the Dutch cervical cancer screening programme: the role of personal and organisational characteristics. Prev Med Rep. 2021;22:101328. doi:10.1016/j.pmedr.2021.101328 33680719 PMC7930587

[9] Castanon A , Green LI , Sasieni P . Impact of screening between the ages of 60 and 64 on cumulative rates of cervical cancer to age 84y by screening history at ages 50 to 59: a population‐based case‐control study. Prev Med. 2021;149:106625. doi:10.1016/j.ypmed.2021.106625 34019928 PMC8223500

[10] Lönnberg S , Anttila A , Luostarinen T , Nieminen P . Age‐specific effectiveness of the Finnish cervical cancer screening programme. Cancer Epidemiol Biomarkers Prev. 2012;21(8):1354‐1361. doi:10.1158/1055-9965.EPI-12-0162 22665576

[11] Lönnberg S , Nieminen P , Luostarinen T , Anttila A . Mortality audit of the Finnish cervical cancer screening program. Int J Cancer. 2013;132(9):2134‐2140. doi:10.1002/ijc.27844 22987437

[12] Pankakoski M , Anttila A , Sarkeala T , Heinävaara S . Effectiveness of cervical cancer screening at age 65—a register‐based cohort study. PLoS One. 2019;14(3):e0214486. doi:10.1371/journal.pone.0214486 30913262 PMC6435141

[13] Schouten L , Botha H , Paci E . Method of Detection in Relation to Screening. March 2001. Accessed November 30, 2024. https://www.encr.eu/sites/default/files/pdf/detection.pdf

[14] Olthof EMG , Aitken CA , Siebers AG , van Kemenade FJ , de Kok IMCM . The impact of loss to follow‐up in the Dutch organised HPV‐based cervical cancer screening programme. Int J Cancer. 2024;154(12):2132‐2141. doi:10.1002/ijc.34902 38436201

[15] World Health Organization (WHO) . Cervical Cancer. World Health Organization; 2024. Accessed June 26, 2024. https://www.who.int/health-topics/cervical-cancer#tab=tab_1

[16] Muñoz N , Bosch FX , De Sanjosé S , et al. Epidemiologic classification of human papillomavirus types associated with cervical cancer. N Engl J Med. 2003;348(6):518‐527. doi:10.1056/NEJMoa021641 12571259

[17] Walboomers JMM , Jacobs MV , Manos MM , et al. Human papillomavirus is a necessary cause of invasive cervical cancer worldwide. J Pathol. 1999;189(1):12‐19. doi:10.1002/(SICI)1096-9896(199909)189:1<12::AID-PATH431>3.0.CO;2-F 10451482

[18] Ronco G , Dillner J , Elfström KM , et al. Efficacy of HPV‐based screening for prevention of invasive cervical cancer: follow‐up of four European randomised controlled trials. Lancet. 2014;383(9916):524‐532. doi:10.1016/S0140-6736(13)62218-7 24192252

[19] Gilham C , Sargent A , Kitchener HC , Peto J . HPV testing compared with routine cytology in cervical screening: long‐term follow‐up of ARTISTIC RCT. Health Technol Assess. 2019;23(28):1‐44. doi:10.3310/hta23280 PMC6600121 31219027

[20] Vahteristo M , Leinonen MK , Sarkeala T , Anttila A , Heinävaara S . Similar effectiveness with primary HPV and cytology screening – long‐term follow‐up of randomized cervical cancer screening trial. Gynecol Oncol. 2024;180:146‐151. doi:10.1016/j.ygyno.2023.11.036 38091774

[21] Fritz A , Percy C , Jack A , et al. International Classification of Diseases for Oncology, 3rd Edition (ICD‐O‐3). Accessed July 8, 2024. https://www.who.int/standards/classifications/other‐classifications/international‐classification‐of‐diseases‐for‐oncology

[22] Leinonen MK , Miettinen J , Heikkinen S , Pitkäniemi J , Malila N . Quality measures of the population‐based Finnish Cancer Registry indicate sound data quality for solid malignant tumours. Eur J Cancer. 2017;77:31‐39. doi:10.1016/j.ejca.2017.02.017 28350996

[23] Veijalainen O , Kares S , Kujala P , et al. Implementation of HPV‐based cervical cancer screening in an organised regional screening programme: 3 years of experience. Cytopathology. 2019;30(2):150‐156. doi:10.1111/cyt.12652 30421573

[24] Finnish Cancer Registry . Kohdunkaulasyövän seulontaohjelma. Accessed July 1, 2024. https://stats.cancerregistry.fi/joukkistilastot/kohtu.html

[25] Muggeo VMR . segmented: Regression Models with Break‐Points/Change‐Points Estimation (with Possibly Random Effects). October 25, 2024. Accessed December 2, 2024. https://cran.r-project.org/web/packages/segmented/index.html

[26] Clegg LX , Hankey BF , Tiwari R , Feuer EJ , Edwards BK . Estimating average annual per cent change in trend analysis. Stat Med. 2009;28(29):3670‐3682. doi:10.1002/sim.3733 19856324 PMC2843083

[27] Petkeviciene J , Ivanauskiene R , Klumbiene J . Sociodemographic and lifestyle determinants of non‐attendance for cervical cancer screening in Lithuania, 2006–2014. Public Health. 2018;156:79‐86. doi:10.1016/j.puhe.2017.12.014 29408192

[28] Tekalegn Y , Sahiledengle B , Woldeyohannes D , et al. High parity is associated with increased risk of cervical cancer: systematic review and meta‐analysis of case–control studies. 18:17455065221075904. doi:10.1177/17455065221075904 PMC8819811 35114865

[29] Deacon JM , Evans CD , Yule R , et al. Sexual behaviour and smoking as determinants of cervical HPV infection and of CIN3 among those infected: a case–control study nested within the Manchester cohort. Br J Cancer. 2000;83(11):1565‐1572. doi:10.1054/bjoc.2000.1523 11076670 PMC2363425

[30] Stelzle D , Tanaka LF , Lee KK , et al. Estimates of the global burden of cervical cancer associated with HIV. Lancet Glob Health. 2021;9(2):e161‐e169. doi:10.1016/S2214-109X(20)30459-9 33212031 PMC7815633

[31] Gargiulo Isacco C , Balzanelli MG , Garzone S , et al. Alterations of vaginal microbiota and chlamydia trachomatis as crucial co‐causative factors in cervical cancer genesis procured by HPV. Microorganisms. 2023;11(3):662. doi:10.3390/microorganisms11030662 36985236 PMC10053692

[32] Asthana S , Busa V , Labani S . Oral contraceptives use and risk of cervical cancer—a systematic review & meta‐analysis. Eur J Obstet Gynecol Reprod Biol. 2020;247:163‐175. doi:10.1016/j.ejogrb.2020.02.014 32114321

[33] Malevolti MC , Lugo A , Scala M , et al. Dose‐risk relationships between cigarette smoking and cervical cancer: a systematic review and meta‐analysis. Eur J Cancer Prev. 2023;32(2):171‐183. doi:10.1097/CEJ.0000000000000773 36440802

[34] Pankakoski M , Heinävaara S , Anttila A , Sarkeala T . Differences in cervical test coverage by age, socioeconomic status, ethnic origin and municipality type – a nationwide register‐based study. Prev Med. 2020;139:106219. doi:10.1016/j.ypmed.2020.106219 32693176

[35] Dunne EF , Unger ER , Sternberg M , et al. Prevalence of HPV infection among females in the United States. JAMA. 2007;297(8):813‐819. doi:10.1001/jama.297.8.813 17327523 10.1001/jama.297.8.813

[36] Forman D , de Martel C , Lacey CJ , et al. Global burden of human papillomavirus and related diseases. Vaccine. 2012;30(5):F12‐F23. doi:10.1016/j.vaccine.2012.07.055 23199955

[37] Falah‐Hassani K , Kosunen E , Shiri R , Jokela J , Liinamo A , Rimpelä A . Adolescent sexual behavior during periods of increase and decrease in the abortion rate. Obstet Gynecol. 2009;114(1):79‐86. doi:10.1097/AOG.0b013e3181a99ddd 19546762

[38] Laukkanen P , Koskela P , Pukkala E , et al. Time trends in incidence and prevalence of human papillomavirus type 6, 11 and 16 infections in Finland. J Gen Virol. 2003;84(pt 8):2105‐2109. doi:10.1099/vir.0.18995-0 12867641

[39] Khandekar MJ , Cohen P , Spiegelman BM . Molecular mechanisms of cancer development in obesity. Nat Rev Cancer. 2011;11(12):886‐895. doi:10.1038/nrc3174 22113164

[40] Vuorela N , Saha MT , Salo MK . Change in prevalence of overweight and obesity in Finnish children – comparison between 1974 and 2001. Acta Paediatr. 2011;100(1):109‐115. doi:10.1111/j.1651-2227.2010.01980.x 20712840

[41] Hassani KF , Kosunen E , Rimpelä A . The use of oral contraceptives among Finnish teenagers from 1981 to 2003. J Adolesc Health. 2006;39(5):649‐655. doi:10.1016/j.jadohealth.2006.05.022 17046500

[42] Guo C , Zhan B , Li MY , Yue L , Zhang C . Association between oral contraceptives and cervical cancer: a retrospective case‐control study based on the National Health and nutrition examination survey. Front Pharmacol. 2024;15:1400667. doi:10.3389/fphar.2024.1400667 39086392 PMC11288899

[43] Nieminen P , Anttila A , Butzow R , et al. Kohdunkaulan, emättimen ja ulkosynnytinten solumuutokset – diagnostiikka, hoito ja seuranta (in Finnish). Duodecim. 2006;122:1808‐1833. 17091721

[44] Horn J , Denecke A , Luyten A , et al. Reduction of cervical cancer incidence within a primary HPV screening pilot project (WOLPHSCREEN) in Wolfsburg, Germany. Br J Cancer. 2019;120(10):1015‐1022. doi:10.1038/s41416-019-0453-2 30988395 PMC6734660

[45] Salo H , Leino T , Kilpi T , et al. The burden and costs of prevention and management of genital disease caused by HPV in women: a population‐based registry study in Finland. Int J Cancer. 2013;133(6):1459‐1469. doi:10.1002/ijc.28145 23463194

[46] Pankakoski M , Sarkeala T , Anttila A , Heinävaara S . Effectiveness of cervical testing in and outside a screening program—a case‐control study. Cancer. 2022;14(21):5193. doi:10.3390/cancers14215193 PMC9653595 36358612

[47] Statistics Finland (Tilastokeskus) . Population 31.12. by Year, Sex, Age and Information. Population structure. Accessed September 8, 2024. https://pxdata.stat.fi:443/PxWebPxWeb/pxweb/en/StatFin/StatFin__vaerak/statfin_vaerak_pxt_11rd.px/

[48] Lynge E , Lönnberg S , Törnberg S . Cervical cancer incidence in elderly women‐biology or screening history? Eur J Cancer. 2017;74:82‐88. doi:10.1016/j.ejca.2016.12.021 28335890

[49] Salo H , Nieminen P , Kilpi T , et al. Divergent coverage, frequency and costs of organised and opportunistic Pap testing in Finland. Int J Cancer. 2014;135(1):204‐213. doi:10.1002/ijc.28646 24347441

[50] Anttila A , Arbyn M , de Vuyst H , et al., eds. European Guidelines for Quality Assurance in Cervical Cancer Screening: Second Edition: Supplements. Publications Office; 2015. doi:10.2875/93363

