# Supplementary material for: Increased cervical cancer incidence in the target age of screening—Variation by mode of detection
Source: Int J Cancer. 2026 Feb 7;159(2):359–67. doi: 10.1002/ijc.70371 (PMC13193351; doi:10.1002/ijc.70371)

Increased cervical cancer incidence in the target age of screening – variation by mode of detection

Jenna Snellman, Maiju Pankakoski, Sirpa Heinävaara, Veli-Matti Partanen, Maija Vahteristo

Supplementary tables 2
Table S1 2
Supplementary figures 3
Figure S1 3

Supplementary tables

Table S1

Table S1. Annual percentage change (APC) estimates and 95% confidence intervals for cervical cancer incidence rates over time.

Overall cancer incidence	Estimated annual percentage change	CI (95%) Lower limit	CI (95%) Upper limit
1996-2007	-1,72	-2,87	-0,57
2008-2022	1,65	0,84	2,46
Squamous cell cancer			
1996-2009	-2,19	-3,35	-1,03
2010-2022	2,63	1,33	3,94
Adenocarcinoma			
1996-2022	0,37	-0,37	1,11
Other			
1996-2022	1,30	-0,18	2,77
At screening age			
1996-2009	1,34	0,52	2,16
Under the screening age			
1996-2022	-0,53	-2,51	1,43
Over the screening age			
1996-2022	-2,95	-3,84	-2,06
Non-attender			
1996-2022	2,02	1,09	2,95
Interval case			
1996-2022	0,86	-1,32	3,06
Screen-detected			
1996-2012	-0,68	-4,14	2,77
2013-2022	10,27	6,02	14,53
No invitation recorded			
1996-2022	-7,37	-8,78	-5,96

Supplementary figures

Figure S1

Fig. S1. The proportion of HPV tests in the screening programme in 1996-2022.

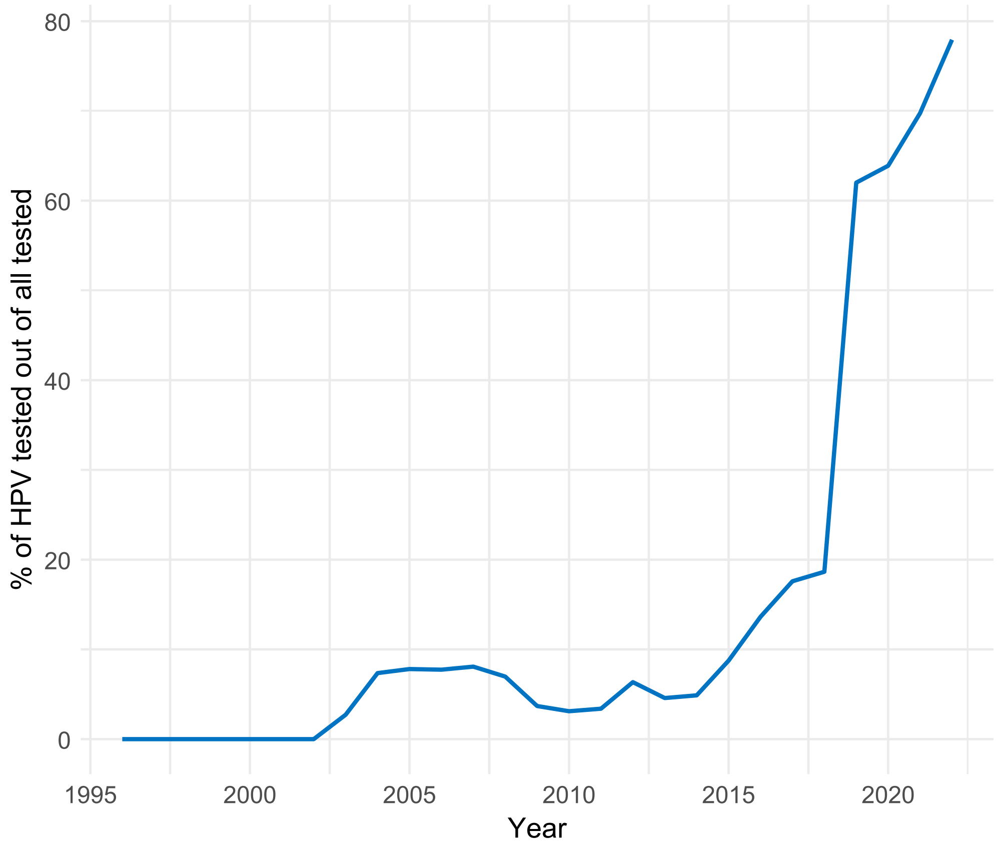

Supplement: Supplementary file 1 — Table S1. Annual percentage change (APC) estimates and 95% confidence intervals. Figure S1. The proportion of HPV tests in the screening program in 1996–2022. [file IJC-159-359-s001.pdf]
